# Uterine rupture in a teaching hospital in Mbarara, western Uganda, unmatched case- control study

**DOI:** 10.1186/1742-4755-10-29

**Published:** 2013-05-29

**Authors:** Peter K Mukasa, Jerome Kabakyenga, Jude K Senkungu, Joseph Ngonzi, Monica Kyalimpa, Van J Roosmalen

**Affiliations:** 1 Ministry of Health, 7272, Kampala, Uganda; 2 UNFPA, Uganda Country office, P.O. BOX 7184, Kampala, Uganda; 3 Mbarara University of Science and Technology, Faculty of Medicine, P.O. BOX 1410, Mbarara, Uganda; 4 School of Nursing Sciences, Kampala International University, Western Campus, P.O. BOX, 71, Ishaka-Bushenyi, Uganda; 5 Department of Obstetrics, Leiden University Medical Centre, Leiden, Amsterdam, the Netherlands; 6 Department of Medical Humanities, EMGO Institute of Health and Care Research, VU University Medical Centre, Amsterdam, the Netherlands

**Keywords:** Uterine rupture, Maternal morbidity, Obstructed labour, Fetal outcome, Obstetric fistula, (previous) Caesarean section, Prolonged labour

## Abstract

**Background:**

Uterine rupture is one of the most devastating complications of labour that exposes the mother and foetus to grave danger hence contributing to the high maternal and perinatal mortality and morbidity in Uganda. Every year, 6000 women die due to complications of pregnancy and childbirth, uterine rupture accounts for about 8% of all maternal deaths.

The objective of this study was to establish the incidence of uterine rupture, predisposing factors, maternal and fetal outcomes and modes of management at a regional referral university hospital in South-western Uganda.

**Methods:**

Case–control design of women with uterine rupture during 2005–2006. Controls were women who had spontaneous vaginal delivery or were delivered by caesarean section without uterine rupture as a complication. For every case, three consecutive in-patient chart numbers were picked and retrieved as controls. All available case files, labour ward and theater records were reviewed.

**Results:**

A total of 83 cases of uterine rupture out of 10940 deliveries were recorded giving an incidence of uterine rupture of 1 in 131 deliveries. Predisposing factors for uterine rupture were previous cesarean section delivery(OR 5.3 95% CI 2.7-10.2), attending < 4 antenatal visits (OR 3.3 95% CI 1.6-6.9), parity ≥ 5(OR 3.67 95% CI 2.0-6.72), no formal education (OR 2.0 95% CI 1.0-3.9), use of herbs (OR15.2 95% CI 6.2-37.0), self referral (OR 6.1 95% CI 3.3-11.2) and living in a distance >5 km from the facility (OR 10.86 95% CI 1.46-81.03). There were 106 maternal deaths during the study period giving a facility maternal mortality ratio of 1034 /100,000 live births, there were 10 maternal deaths due to uterine rupture giving a case fatality rate of 12%.

**Conclusion:**

Uterine rupture still remains one of the major causes of maternal and newborn morbidity and mortality in Mbarara Regional referral Hospital in Western Uganda. Promotion of skilled attendance at birth, use of family planning among those at high risk, avoiding use of herbs during pregnancy and labour, correct use of partograph and preventing un necesarry c-sections are essential in reducing the occurences of uterine repture.

## Background

Worldwide, every year, between 340,000 and half a million women die due to complications of pregnancy and child birth, the majority of these occurring in low income countries. Sub- Sahara Africa bears over 90 percent of the burden [1-3].

Uterine rupture, one of the major obstetric complications of labour contributes significantly to maternal and perinatal mortality and morbidity [4-8]. The occurrence of uterine rupture varies in different parts of the world. While it is rare in high-income countries, it remains a public health problem in low income countries, particularly in Africa and mainly occurring as consequence of prolonged, obstructed labour [6-8].

Uganda like any other country in Sub-Sahara Africa still struggles with poor reproductive health indicators. Every year, 6000 women die due to complications of pregnancy and childbirth, uterine rupture accounts for about 8% of all maternal deaths [5,9-13]. A high incidence of uterine rupture is an indicator of poor obstetric care, poor accessibility to the few available comprehensive Emergence Obstetric Care (EmoC) facilities as well as a poor socio-economic condition of the community [14-18].

In high income countries, the majority of cases occur in women with previous caesarean section, while in low income countries, it usually results from prolonged obstructed labour, often in unscarred uterus. However, most cases are usually associated with a combination of risk factors including grand multiparty, advanced age, fetal macrosomia and abnormal placentation [6-8,19,20].

This study aimed at establishing the incidence of uterine rupture, predisposing factors, maternal and fetal outcomes and modes of management at a regional referral university hospital in South-western Uganda.

## Materials and methods

### Study setting

The study setting was Mbarara University of Science and Technology teaching hospital which is located in Mbarara Municipality, and 286 km south west of Kampala the Capital city of Uganda. It is a public hospital funded by Government of Uganda through the Ministry of Health. It is the referral hospital for south western Uganda serving 10 districts with a population of more than 2.5 million people. It also receives patients from neighbouring countries of Rwanda, Tanzania and Democratic Republic of Congo. It handles on average 10,000 deliveries per year.

### Case definition and selection

Uterine rupture was defined as tearing of the uterine wall either partially or complete during pregnancy and labour, diagnosed either clinically and later confirmed at laparotomy. The cases were retrospectively collected from the maternity ward and operating theatre registers as well as from the patients’ case files at the hospital medical records office.

### Selection of controls

Controls were women who had spontaneous vaginal delivery or were delivered by caesarean section without uterine rupture as a complication. For every case, three consecutive in-patient chart numbers were picked and retrieved as controls.

### Data collection

Data was abstracted from the maternity ward and operating theatre registers as well as from the patients’ case files at the hospital medical records office using a pre tested case report form (CRF). Information on the patients’ age, tribe, address, occupation, religion, parity, previous caesarean section, antenatal care attendance, estimated distance of residence from the hospital, place of intrapartum care, subsequent rupture, type of surgical intervention (total or sub-total hysterectomy, repair without bilateral tubal ligation [BTL] or repair with BTL), maternal and foetal outcomes, length of postoperative hospital stay and other relevant information were collected. The total number of cases of uterine rupture and deliveries from the maternity ward admission register was validated with the annual Health Management Information System (HMIS) reports.

The data collectors were midwives who were trained to collect data from women’s obstetric files or charts and to validate the diagnosis of obstructed labour using admission, delivery and theatre registers. Thought the data collection period which lasted 4 weeks, I was providing oversight and supervision to prevent under reporting.

### Statistical analysis

The data were entered and analyzed using SPSS statistical software, version 12.0 (SPSS, Chicago, IL, USA). Descriptive statistics were obtained through frequencies and cross tabulations. Comparison between groups was made using the x^2^ tests and Fisher exact test when appropriate. All analyses were two-tailed and the level of significance was set at 5%.

### Ethical considerations

Ethical approval was obtained from Mbarara University Institutional Ethical Research Committee. Permission to access obstetric records was obtained from the medical director of the hospital and these were anonymously entered into the database.

## Results

Between January 2005 and December 2006, there were 10,940 deliveries, 10246 live births, 694 still births, giving a stillbirth rate of 68 per 1000 live births and 106 maternal deaths, giving a facility ratio of 1035 per 100,000 live births.

Eight three cases of uterine ruptures were managed during the study period, giving an overall incidence rate of 0.76% or 1 in 131 deliveries. There were 10 maternal deaths due to uterine rupture giving a case fatality rate of 12%. Fresh stillbirths occurred in 80.5% of the cases. Only 77 out of 83 charts were recovered from the registry and hence analyzed for this study. We had to exclude 44 (17%) of the control charts for lack of insufficient information.

75.3% of the cases were in the 20–34 age range which is similar to 75.5% in the 20–34 age range of the controls. The lowest incidence of uterine rupture was among the less than 19 year old age group.

The majority of the women 74% versus 82% attended antenatal care, and mostly getting care from health centres. Those who used herbs were 35.1% among the cases as compared to only 3.4% among the controls [Table 1].

**Table 1:** Demographic characteristics of study participants

**Characteristics**	**Cases**	**Controls**
	**n = 77(%)**	**n = 205(%)**
**Age group**		
≤ 19 years	5(6.5)	32(15.6)
20-34 years	58(75.3)	153(72.5)
≥ 35 years	14(18.2)	20(9.8)
Married	72(93.5)	194(94.6)
Secondary education and above	15(19.5)	68(33.2)
Para 5 and above	29(37.7)	29(14.1)
Living within a distance of 5 km from health facility	1(1.3)	26(12.9)
ANC attendance	57(74)	168(82)
**Place of antenatal**		
Hospital	13(22.9)	69(41.1)
Health centre	42(73.7)	87(51.8)
TBA	1(1.8)	3(1.8)
Referred from facility	38(36.4)	28(14.0)
Partograph used during labour	1(1.3)	42(20.5)
Previous caesarean section	28(36.4)	20(9.8)
Use of herbs in during labour	27(35.1)	7(3.4)

*ANC* Antenatal care, *TBA* Traditional birth attendant.

Predisposing factors for uterine rupture were previous caesarean section (OR 5.3; 95% CI 2.7-10.2), attending <4 antenatal visits (OR 3.3; 95% CI 1.6-6.9), parity ≥5(OR 3.67; 95% CI 2.0-6.72), no formal education (OR 2.0; 95% CI 1.0-3.9), use of herbs (OR 15.2; 95% CI 6.2-37.0), self referral (OR 6.1; 95% CI 3.3-11.2) and living in a distance >5 km from the facility (OR 10.86; 95% CI 1.46-81.03), lack of partograph use (OR 19.57; 95% CI 2.65-144.8) and referral from facility(OR 6.14; 95% CI 3. 37–11.2) [Table 2].

**Table 2:** Associated risk factors for uterine ruptures among study participants

**Variable**	**Cases**	**Controls**	**Odds ratio (CI)**	**p-value**
	**n = (%)**	**n = (%)**		
**Previous caesarean section**				
Yes	28(36)	20(10)	5.3(2.7-10.2)	0.00
No	49(64)	185(90.2)		
**Attended antenatal care(ANC)**				
Yes	57(74)	168(82)	0.62(0.33-1.2)	0.142
No	20(26)	37(18)		
**Number of visits attended for antenatal care**				
< 4 times	47(82.5)	99(58.9)	3.3(1.6-6.9)	0.002
≥4 times	10(17.5)	6941.1)		
**Parity**				
≥ 5	29(38)	29(14)	3.67(2.0-6.72)	0.000
≤ 4	48(62)	176(86)		
**Education level**				
None or primary	62(81)	137(67)	2.05(1.09-3.87)	0.026
Secondary and above	15(19)	68(33)		
**Partograph use**				
No	76(98.7)	163(79.5)	19.57(2.65-144.8)	0.004
yes	1(1.3)	42(20.5)		
**Use of herbs**				
Yes	27(35)	7(3)	15.27(6.2-37.09)	0.000
no	50(65)	198(97)		
**Referred**				
From facility	38(49)	33(16)	6.14(3.37-11.2)	0.000
self	39(51)	172(84)		
**Distance travelled from home to facility**				
More than 5 KMs	76(99)	176(86)	10.86(1.46-81.03)	0.02
Within 5 KMs	1(1.3)	29(14)		

Total abdominal hysterectomy was done in 22 (28.6%) women with uterine rupture, subtotal hysterectomy in 29 (37.7%), uterine repair with BTL in 4 (5.2%) and uterine repair without BTL in 22 (28.6%) [Table 3].

**Table 3:** Types of operations done

**Operation procedure**	**N (%)**
Total abdominal hysterectomy	22(28.6)
Total abdominal hysterectomy	29(37.7)
Repair of the uterus	22(28.6)
Repair of the uterus and bilateral tubal ligation	4(5.2)

## Discussion

The incidence of uterine rupture in this study was 1in 131 deliveries (0.76%), which is slightly higher than other recent studies done in Uganda in which the incidence was 1 in 200 deliveries. This shows that uterine rupture contributes greatly to the maternal and new born morbidity and mortality in western Uganda and is a reflection of the flaws in the health system in the region [5,10,21,22]. Other studies in sub-Saharan Africa have also reported almost similar results as compared to 1 in 1536 (0.07%) in high income countries where there is a wider availability and utilisation of medical services is [6-8,19,23-26].

For this study period, there were 106 maternal deaths in the hospital and 10 of these where due to uterine rupture giving a facility maternal mortality ratio of 1034 per 100,000 live births and a case facility rate of 12%, which is well above the less than 1% fatality rate recommended by World Health Organisation [27]. This could be compared with other studies done from developing countries and explained by the enormous challenges of access for quality services highlighted in the 3 delays model by Maine, including delays of reaching facilities, our facility being a regional referral, women often come in critical condition resulting in poor outcomes and in developed countries, the case fatality ranges between 0-1% [27,28].

Only 19.5% live births were recorded among the mothers with uterine rupture, the majority (16.2%) were from scar uterine rupture, which is in the range reported in some countries [15,19,29-37]. To avoid these high mortalities, there is need to put in place systems for timely diagnosis, stabilization and interventions [25]. All these finding show important operational deficiencies in the provision of obstetric care in Uganda [26].

Despite high use of antenatal care, over 94% for first visit in Uganda, deliveries at health facilities has remained low 42%, Emergency Obstetric Care met need is only 14%. Postnatal care (PNC) coverage is very low and maternal mortality, perinatal and neonatal mortality have remained high at 435 per 100,000 live births, 36 per 1,000 live births, 29 per 1000 live births respectively [11,38,39].

In this current study, over 82% of the cases attended antenatal care less than the recommended 4 times and were 3 times more likely to sustain uterine rupture and these are likely to deliver at home with no birth preparedness compared to those who attended 4 times, such results have been reported in other studies in Ethiopia and Nigeria [17,34,35].

Majority of the cases (64%) and controls (90.2%) in this study had no history of previous caesarean section or operation on the uterus which is in agreement with a study from Nigeria and other sub Saharan countries as most of the uterine ruptures are associated with obstructed labour. Those who had a history of previous caesarean section were 5 times more likely to sustain uterine rupture and this in agreement with other studies especially in high income countries, this could be explained that there could have been some degree of post partum infection and hence weaken the scar [7,20,36,37].

Many have considered multiparity as a risk factor for uterine rupture, in this study, those with parity ≥5 were four times likely to get uterine rupture. This could be explained that in women with less parity and especially in primigravidas, when mechanical obstruction occurs, uterine contractions gradually become weaker and stop, while in multi-parious women, the contractions often continue till delivery and end up with uterine rupture [17,21].

Most studies have indicated that lower socio economic status in combination with lack of education are associated with poor health seeking behaviour and access to care, hence resulting into high proportions of lack of skilled attendance at birth, prolonged labour and uterine rupture [34-38]. In this study, women with no education or those who ended in primary education were two times more likely to sustain uterine rupture and this in agreement with other studies that found that women with no formal education were more prone to no use of antenatal care, skilled birth attendance, postnatal care and use of family planning methods [39-46].

Appropriate use of the partograph is an important tool for audit and monitoring progress of labour and a warning device to detect deviations from normal labour, preventing obstructed labour and thereby improving maternal and fetal outcome [47-49]. However, its use has been a challenge in most facilities probably due to lack of skills, negative attitudes by service providers and lack of papers. Not using a partograph was 19 times more likely to result in uterine rupture. All these factors have to be considered in planning for implementation of proper use of partographs in health settings [47].

Use of herbs as complementary medicines during pregnancy and labour is common in our setting and is compounded by many cultural and traditional briefs [49,50]. These herbs are thought to have oxytoxic ingredients and those who take them during labour tend to get hyper stimulation of the uterus leading to uterine rupture and fetal hypoxia and demise. In this study, those who used herbs during labour were 15 times more likely to get uterine rupture than who did not.

Ministry of Health recommends that for easy access to emergency obstetric care, the community is served by a health facility which is in a distance of less than 5 kms. In this study, all the mothers came from a distance far more than 5 kms and they were 10 times more likely to get uterine rupture due long distances, delays to reach and poor road network [11-13].

Proper diagnosis is paramount and this is based on clinical signs and symptoms of uterine rupture. Proper stabilization of the patient before surgery is critical as this improves outcome and prognosis. Modes of management of uterine rupture will be based on the extent of rupture, desire of mother, the number of children she has, the decision and experience of the physician on the operating table in theatre. Modes of management include total hysterectomy, subtotal hysterectomy [8,15,19,29-35], repair with bilateral and repair without tubal ligation. However, there is need to get informed consent for sterilization from the patient or couple [51,52].

### Study limitations

This being a facility based study; the findings may not be generalised to the general population. Due to the retrospective nature of the study, much information was missing in the charts and maternity registers and hence a great possibility of underreporting. May be the small sample size of the study may have limited our ability to detect small differences.

### Implications for practice

There is a need to address third delay at the health facilities since facility level preparedness to respond to obstetric and newborn emergencies is critical for the survival of women and their newborns. Health facilities especially at primary level need to be supported with adequate skilled birth attendants, equipment, drugs and supplies for appropriate care during pregnancy and child birth. Government of Uganda needs to implement the Road Map which has been developed to reach the rural woman and sensitise them on birth preparedness, skilled attendance, and safe motherhood and empower them with knowledge to seek health care. Since over 90% of women attend antenatal care at least once during pregnancy, there is a need to utilize this opportunity to counsel women and their husbands if available on birth preparedness In addition, partographs should be available in all facilities and used correctly.

## Conclusions

Uterine rupture still remains one of the major causes of maternal and newborn morbidity and mortality in Mbarara Regional referral Hospital in Western Uganda. Promotion of skilled attendance at birth, use of family planning among those at high risk, avoiding use herbs during pregnancy and labour, correct use of partograph and preventing un necesarry c-sections are essential in reducing the occurences of uterine repture.

## Competing interests

The authors declare that they have no competing interests.

## Authors’ contributions

PKM, JK, MK, VRM conceived and designed the study, developed data collection instruments and supervised data collection. PKM, JS, JN, MK participated in the testing and finalization of the data collection instruments and coordinated study progress JS, PKM, JK performed the statistical analysis, PKM wrote all versions of the manuscript.VRM gave in put during statistical analysis mentored PKM on writing all the versions and critically reviewed all the versions. All authors read and approved the final manuscript.
